# Transcriptional Regulation of *MECP2E1-E2* Isoforms and *BDNF* by Metformin and Simvastatin through Analyzing Nascent RNA Synthesis in a Human Brain Cell Line

**DOI:** 10.3390/biom11081253

**Published:** 2021-08-22

**Authors:** Marjorie Buist, David Fuss, Mojgan Rastegar

**Affiliations:** Department of Biochemistry and Medical Genetics, Rady Faculty of Health Sciences, Max Rady College of Medicine, University of Manitoba, Winnipeg, MB R3E 0J9, Canada; buistm@myumanitoba.ca (M.B.); fussd@myumanitoba.ca (D.F.)

**Keywords:** DNA methylation, MeCP2, MeCP2 isoforms, *MECP2E1*, *MECP2E2*, Rett Syndrome, metformin, statins, simvastatin, *BDNF*, ribosomal RNA, transcription, Daoy medulloblastoma cells

## Abstract

Methyl CpG binding protein 2 (MeCP2) is the main DNA methyl-binding protein in the brain that binds to 5-methylcytosine and 5-hydroxymethyl cytosine. *MECP2* gene mutations are the main origin of Rett Syndrome (RTT), a neurodevelopmental disorder in young females. The disease has no existing cure, however, metabolic drugs such as metformin and statins have recently emerged as potential therapeutic candidates. In addition, induced *MECP2-BDNF* homeostasis regulation has been suggested as a therapy avenue. Here, we analyzed nascent RNA synthesis versus steady state total cellular RNA to study the transcriptional effects of metformin (an anti-diabetic drug) on *MECP2* isoforms (*E1* and *E2*) and *BNDF* in a human brain cell line. Additionally, we investigated the impact of simvastatin (a cholesterol lowering drug) on transcriptional regulation of *MECP2E1/E2-BDNF*. Metformin was capable of post-transcriptionally inducing *BDNF* and/or *MECP2E1*, while transcriptionally inhibiting *MECP2E2*. In contrast simvastatin significantly inhibited *BDNF* transcription without significantly impacting *MECP2E2* transcripts. Further analysis of ribosomal *RNA* transcripts confirmed that the drug neither individually nor in combination affected these fundamentally important transcripts. Experimental analysis was completed in conditions of the presence or absence of serum starvation that showed minimal impact for serum deprival, although significant inhibition of steady state *MECP2E1* by simvastatin was only detected in non-serum starved cells. Taken together, our results suggest that metformin controls *MECP2E1/E2-BDNF* transcriptionally and/or post-transcriptionally, and that simvastatin is a potent transcriptional inhibitor of *BDNF*. The transcriptional effect of these drugs on *MECP2E1/E2-BDNF* were not additive under these tested conditions, however, either drug may have potential application for related disorders.

## 1. Introduction

Rett Syndrome (RTT) is a rare and progressive neurodevelopmental disorder that results from *de novo* mutations in the X-linked methyl-CpG binding protein 2 (*MECP2*) gene [1]. RTT has no available cure, and current clinical efforts are mostly toward managing the symptoms. MeCP2 is the main protein in the brain that binds to methylated DNA, in the context of 5-methyl cytosine, known as 5-mC and 5-hydroxymethyl cytosine, commonly referred to as 5-hmC [2]. DNA methylation is a major epigenetic modification that is involved in brain cellular differentiation and neuronal maturation [3,4,5]. As such, deregulated DNA methylation is largely involved in neurodevelopmental disorders [6,7], and has been the subject of intensive investigations [2,8,9,10]. The X-linked *MECP2/Mecp2* gene produces multiple coding and non-coding transcript isoforms, among them, *MECP2E1/Mecp2e1* and *MECP2E2/Mecp2e2* are the best studied splice variants [11,12,13,14,15,16,17]. In the brain, MeCP2E1 is the dominant protein isoform and has direct relevance to Rett Syndrome [18,19,20]. Our team has reported that the murine *Mecp2* gene regulation is coordinated by DNA methylation at the *Mecp2 cis*-regulatory elements in the promoter regions (R1-to-R3) and its first intron (R4-to-R6) in the brain and brain cells [12,21,22,23]. Indeed, certain CpG dinucleotide methylation at R1–R6 are correlated with the expression of *Mecp2*/MeCP2 isoforms (E1 and E2) in the murine brain, and primary embryonic brain cells that include self-renewing and differentiated neural stem cells, astrocytes, and cortical neurons [11,12,21,22,23].

In addition to *MECP2E1* and *E2* isoforms, regulation of the brain-derived neurotrophic factor (*BDNF*) is also important for RTT [24]. BDNF promotes cell survival, neurite outgrowth, synaptic transmission, synaptic plasticity, and cell migration in neurons [25], processes that are defective in RTT and *MECP2* Duplication Syndrome. Recent studies from our team have also highlighted impairment of the *MECP2E1/E2-BDNF* homeostasis in RTT patients that is regulated in a brain region-specific manner [26]. Our immunohistochemical analysis of the human brain tissues from RTT patients have indicated that both neurons and glial cells are important and that RTT patients have compromised cell signaling pathways in the cerebellum [27,28].

As RTT has no cure, identification of potential therapeutic candidate drugs has been a research priority [29,30]. Recent pre-clinical efforts for RTT have been focused on commonly used metabolite drugs that have low or no side-effects. In this respect, statins have been emerging candidate drugs for RTT [31]. Simvastatin is a member of the statin family of drugs used to treat dyslipidemia in patients with elevated cholesterol by inhibiting HMG-CoA reductase, which is a critical step in cholesterol biogenesis. Elevated cholesterol is also observed in RTT patients and mouse models [32,33,34]. Statins have been implicated in providing neuroprotection for various cognitive and neurological disorders [35,36]. Upregulation of BDNF has been observed in mouse models treated with simvastatin following brain injury and spinal cord injury [37]. Another candidate drug is metformin that acts as an mTOR inhibitor [38], a fundamentally important signaling pathway that is impaired in the brain of RTT patients [28]. Metformin is commonly used for treatment of type 2 diabetes patients [39], and has shown promising effects for neurological disorders [40,41,42]. The mechanisms of metformin action in brain cells still require further investigation including whether increases in *MECP2E1/E2* and/or *BDNF* transcription are involved.

Here, we studied the transcriptional effects of metformin and simvastatin on *MECP2E1/E2-BDNF* transcripts through nascent RNA analysis in a human brain cell line. These drugs were selected because of their ability to cross the blood–brain barrier [43,44], their neuroprotective potential in other neurological diseases [45,46,47], and their potential to correct defects in carbohydrate and cholesterol metabolism that have been observed in RTT patients [31]. The focus of this study was to investigate the effects of metformin and simvastatin treatment on *MECP2E1*, *MECP2E2*, *BDNF*, and ribosomal *RNA (rRNA)* transcription in a human brain cell line (Daoy cells). Drugs that induce *MECP2* levels are of interest, since even increased expression of the mutated MeCP2 in *Mecp2*-T158M knock in mice ameliorated symptoms [48]. Induction of *BDNF* is also a desired effect of drug treatment, and has been a targeted molecule for RTT [19].

## 2. Material and Methods

### 2.1. Cell Cultures and Drug Treatments

**Figure 1:**
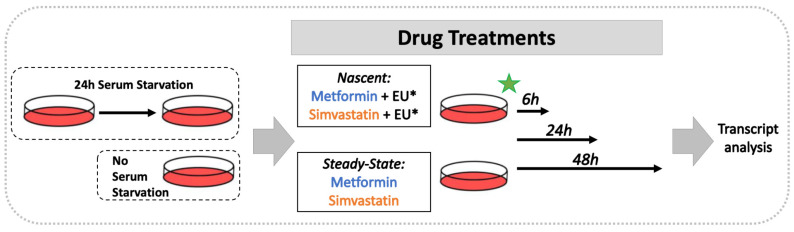
Schematic representation of Daoy cell treatment with metformin and simvastatin.

Daoy cells (ATCC HTB-186) were cultured in MEM with the addition of 10% FBS, 1 mM sodium pyruvate, and 1% penicillin-streptomycin-glutamine (Gibco, Thermo Fisher Scientific, Waltham, MA, USA). Cells were cultured and maintained at 37 °C and 5% CO_2_. Metformin (metformin hydrochloride: catalogue number PHR1084, lot number: LRAA8975 = lot A, lot number: P500240 = lot B, Sigma-Aldrich, Saint Louis, MO, USA) was dissolved in water. All metformin experiments were completed with lot A, except where the data presents a side-by-side comparison of both lots; A and B. Simvastatin (Sigma-Aldrich) was dissolved in DMSO. The final concentration of DMSO applied to simvastatin treated cells did not exceed 0.0125%. Drug treatments were done in conditions with or without prior serum starvation. Cells that underwent serum starvation were cultured in serum-free media for 24 h (h) prior to drug treatment, as shown in Figure 1.

### 2.2. Total RNA Extraction, Nascent RNA Collection, cDNA Preparation, and qRT-PCR

Total RNA extraction was done with RNeasy Plus Mini Kit (Qiagen, Hilden, Germany), according to the manufacturers’ protocols, and as reported [49]. The cDNA synthesis of total RNA was done by Superscript III reverse transcriptase (Invitrogen, Thermo Fisher Scientific, Waltham, MA, USA), as reported previously [21,22].

For nascent RNA, metabolic labelling was followed by RNA isolation for targeted gene-specific transcript analysis. The 4-thiouridine labeled RNAs was purified by biotin/streptavidin interaction [50]. The 5-ethynyl-uridine (EU) was incorporated into RNA transcripts by RNA polymerases I, II, and III, specifically labeling the newly synthesized RNA and labeling approximately one in every 35 uridines. EU was then detected with click chemistry by a copper(I)-catalyzed cycloaddition reaction [51]. Nascent RNA was isolated with Click-iT Nascent RNA Capture Kit (Invitrogen, Thermo Fisher Scientific). Daoy cells were labelled with 0.1 mM 5-ethynyl-uridine in the culture media for, 6 h, 24 h, or 48 h. Cells were harvested, snap frozen, and RNA was extracted with the RNeasy Mini Kit. EU-RNA was then biotinylated by a copper-catalyzed click reaction using an azide-modified biotin and precipitated overnight at −80 °C.

Biotinylated EU-RNA were bound to Dynabeads MyOne Streptavidin T1 magnetic beads according to the manufacturer protocol. We used the Qiagen magnetic tube rack for steps requiring immobilization of the beads. This was followed by cDNA synthesis on the beads. Beads were heated for 5 min at 68–70 °C, followed by the addition of the cDNA synthesis mix containing random primers and dNTPs. The mix was brought to room temperature and subjected to cDNA synthesis by Superscript III Reverse Transcriptase. The reaction was done at 50 °C for 1 h. The reaction was then terminated, and cDNA released from the beads by heating at 85 °C for 5 min. Beads were immobilized and supernatant containing cDNA was collected.

Gene expression analysis was conducted by qRT-PCR on the Applied Biosystems 7500 Fast Real-Time PCR System with PowerUp SYBR Green Master Mix (Applied Biosystems, Thermo Fisher Scientific), as previously reported [52]. The sequences of gene-specific primers are shown in Table 1. Target gene C_T_ values were normalized to the housekeeping gene *GAPDH*. Fold change values were calculated by the 2^−ΔΔCT^ method.

### 2.3. Statistical Analysis

Statistical analysis was done by GraphPad Prism (Version 8.4.0, San Diego, CA, USA), as we previously reported [27,52]. Comparisons between three or more groups were analyzed by one way ANOVA followed by the Tukey multiple comparisons test, with an alpha of 0.05. Levels of significance were determined as * *p* < 0.05, ** *p* < 0.01, *** *p* < 0.001, and **** *p* < 0.0001.

## 3. Results

### 3.1. Metformin Regulates MECP2 and BDNF Transcripts in a Time- and Dose-Dependent Manner

**Figure 2:**
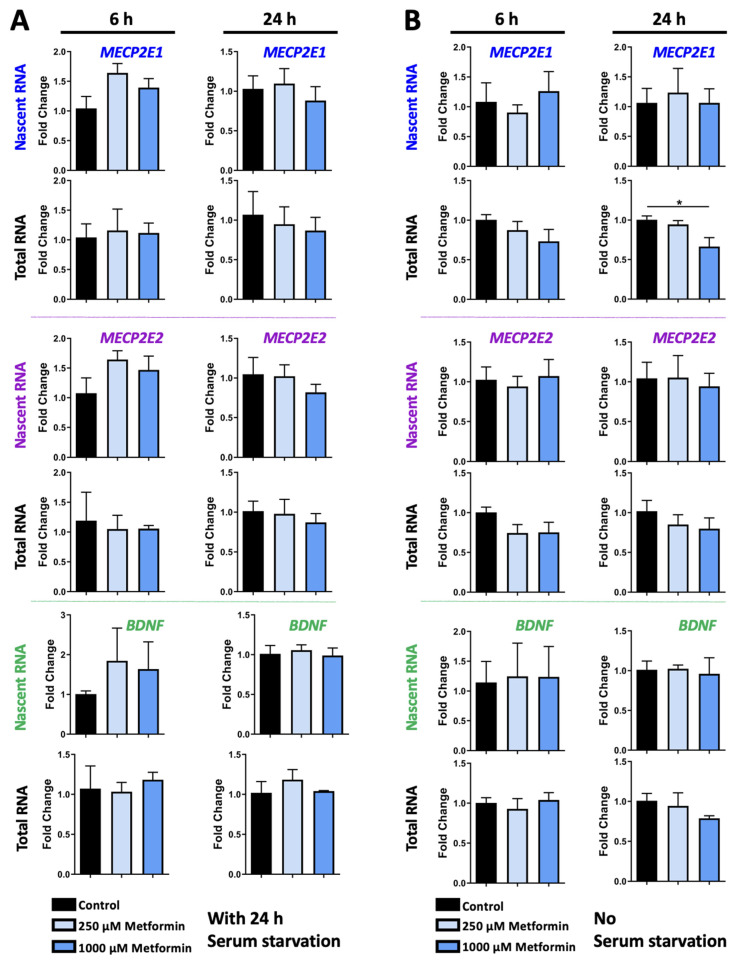
Transcriptional effect of metformin on *MECP2E1-E2* and *BDNF* in Daoy cells under the condition of with and without serum starvation. (**A**) Daoy cells were serum-starved for 24 h prior to drug treatment with 250 and 1000 μM metformin. Samples were collected at 6 and 24 h and gene-specific nascent and steady RNA transcripts were analyzed by RT-PCR for *MECP2E1*, *MECP2E2*, and *BDNF*. C_T_ values were normalized to the housekeeping gene *GAPDH* and fold change was determined relative to the average control. N = 3 ± SEM. Fold change values were analyzed by one-way ANOVA followed by Tukey’s multiple comparisons test, * *p* < 0.05. (**B**) Similar to A, but Daoy cells were subjected to the same drug treatment, without prior serum starvation.

To study the transcriptional role of metformin on *MECP2E1/E2* and *BDNF* transcripts, Daoy cells were treated with two different concentrations of 250 μM and 1000 μM metformin. Cells were exposed to metformin for 6 h and 24 h, and under two conditions of 24 h serum starvation or no serum starvation. Daoy cells were seeded at 50,000 cells per well in 6-well plates. Approximately 16 h later, cells were subjected to serum starvation and cultured in serum-free media for 24 h, followed by drug treatments in media containing serum. One set of drug-treated cells were labelled with 0.1 mM EU to label the nascent RNA produced during drug treatments, and a second set of cells was left unlabeled for analysis of steady-state total RNA transcripts. Based on the Click iT Nascent RNA Capture Kit manufacturer’s recommendations, two time points of 6 h and 24 h were initially selected for RNA collection and analysis. Under these conditions, we did not detect any significant changes in the nascent or steady-state *MECP2E1*, *MECP2E2*, and *BDNF* transcripts (Figure 2A). Under conditions with no serum starvation, *MECP2E1* steady-state transcripts were slightly lowered by about 30% in 1000 μM metformin treated cells at 24 h (*p* < 0.05) (Figure 2B, top panels). There were no other statistically significant changes in *MECP2E2* or *BDNF* transcripts in metformin treated cells (Figure 2B, middle and bottom panels), indicating similar trends to metformin treated cells in the serum starved condition. A slight pattern of decreased *MECP2E1* steady-state transcripts was observed at 6 h, but this was not statistically significant.

**Figure 3:**
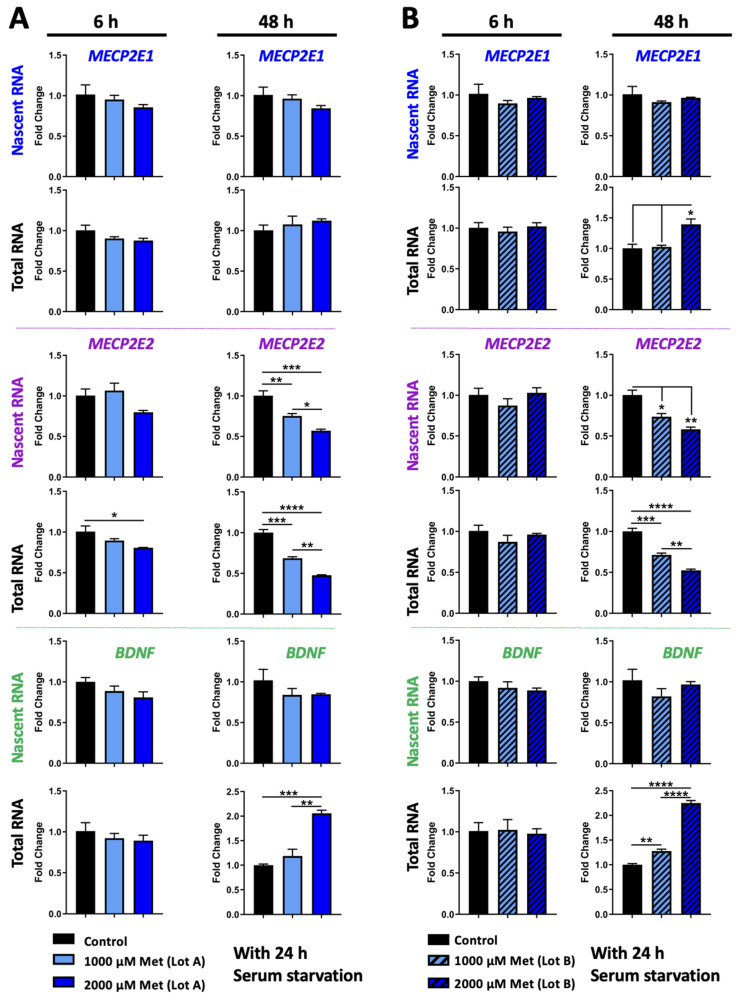
Transcriptional effect of two lots of metformin on *MECP2E1-E2* and *BDNF* in Daoy cells with serum starvation. (**A**,**B**) Daoy cells were serum-starved for 24 h prior to drug treatment with 1000 and 2000 μM metformin (Met) with two lots of the drug from the same company. Samples were collected at 6 and 48 h and gene transcripts were analyzed by RT-PCR for *MECP2E1*, *MECP2E2*, and *BDNF*. C_T_ values were normalized to the housekeeping gene *GAPDH* and fold change was determined relative to the average control. N = 3 ± SEM. Fold change values were analyzed by one-way ANOVA followed by Tukey’s multiple comparisons test, * *p* < 0.05, ** *p* < 0.01, *** *p* < 0.001, and **** *p* < 0.0001.

Next, we asked the question whether increased metformin concentration and additional exposure time may have any impact. We kept cells at the time-point of 6 h and 1000 μM as a common condition with the first set of treatment and extended the treatment time to 48 h. Thus, Daoy cells were treated with 1000 μM and 2000 μM metformin for 6 h and 48 h (Figure 3A). Our results indicated that like 24 h, the nascent and steady-state *MECP2E1* transcripts were still not significantly changed (Figure 3A, top panels). As previously, nascent *MECP2E2* transcripts were unchanged at 6 h but the steady-state level of *MECP2E2* transcripts showed slight but significantly decreased levels by approximately 20% in 6 h 2000 μM treated cells (*p* < 0.05) (Figure 3A, middle panels). After 48 h *MECP2E2* transcripts showed higher levels of significant decrease. The nascent *MECP2E2* transcripts were reduced approximately 25% by 1000 μM (*p* < 0.01) and approximately 40% by 2000 μM treatment (*p* < 0.001). The decrease in nascent *MECP2E2* in 2000 μM treated cells was also significantly lower compared to 1000 μM treated cells (*p* < 0.05), indicating a dose-dependent effect of metformin. Steady-state *MECP2E2* transcripts were decreased approximately 30% by 1000 μM (*p* < 0.001) and approximately 50% by 2000 μM treatment (*p* < 0.0001). The difference between 1000 μM and 2000 μM treatment was also statistically significant (*p* < 0.01). The decrease in *MECP2E2* transcripts at both the nascent and steady-state levels indicated that metformin treatment reduces *MECP2E2* at the transcriptional level. Nascent and steady-state *BDNF* transcripts were unchanged at 6 h (Figure 3A, bottom panels). Nascent *BDNF* transcripts remained unchanged after 48 h, but steady-state *BDNF* transcripts were significantly increased by about 2-fold compared to the controls in cells treated with 2000 μM (*p* < 0.001) at 48 h, which was also significantly greater compared to the cells treated with 1000 μM metformin (*p* < 0.01). The increase in *BDNF* transcripts only at the steady-state level suggested that metformin treatment may impact *BDNF* post-transcriptionally, which may possibly involve altered transcript stability.

In our studies, we noticed some lot variation for metformin with regard to the effects on *MECP2E1* transcripts. Indeed, lot variation has been noted for metformin HCl [58]. Therefore, we completed a new side-by-side experiment to study the effect of Daoy cells with metformin from the same company and catalogue number, but two different lot numbers, the new lot being referred to as Lot B, at concentrations of 1000 μM and 2000 μM for 6 and 48 h (Figure 3B). Our analysis showed some lot-specific differences for metformin only with regard to inducing *MECP2E1* transcripts, while a similar effect was detected for *MECP2E2* and *BDNF*. The levels of nascent and steady-state *MECP2E1* transcripts at 6 h or 48 h of nascent remained unchanged, however, steady-state *MECP2E1* transcripts were increased by 40% in cells treated with 2000 μM metformin for 48 h, which was significant compared to both the control and 1000 μM treated cells (*p* < 0.05) (Figure 3B, top panels). Nascent *MECP2E2* transcripts were again unchanged at 6 h and steady-state *MECP2E2* was also unchanged using this lot number (Figure 3B). However, after 48 h, nascent and steady-state *MECP2E2* transcripts showed similar results to that of metformin lot number A. Nascent *MECP2E2* transcripts were decreased approximately 30% by 1000 μM metformin (*p* < 0.05) and approximately 40% by 2000 μM treatment (*p* < 0.01). Steady-state *MECP2E2* transcripts were reduced by approximately 30% by 1000 μM (*p* < 0.001) and approximately 50% by 2000 μM treatment (*p* < 0.0001). The decrease in steady-state *MECP2E2* in 2000 μM treated cells was again significantly lower compared to 1000 μM treated cells (*p* < 0.01). Nascent and steady-state *BDNF* transcripts were unchanged at 6 h (Figure 3B, bottom panels). Nascent *BDNF* transcripts remained unchanged after 48 h, but steady-state *BDNF* transcripts were significantly increased by about 1.3-fold in cells treated with 1000 μM metformin (*p* < 0.01), a result unique to this specific lot of metformin. Steady-state *BDNF* transcripts were significantly increased in cells treated with 2000 μM metformin by approximately 2.25-fold, which was statistically significant compared to both the control and cells treated with 1000 μM metformin (*p* < 0.0001). Taken together, the results for 1000 and 2000 μM metformin were shown to be largely reproducible with two different lot numbers of metformin. The reduction in *MECP2E2* transcripts at the transcriptional level was reproduced as well as the increase in *BDNF* transcripts at the steady-state level. Our results indicated that both tested lots of metformin transcriptionally inhibited *MECP2E2* transcripts, while induced *BDNF* post-transcriptionally (Figure 3A,B, middle and bottom panels).

### 3.2. Simvastatin Treatment Does Not Impact MECP2E2 but Inhibits BDNF Transcription in Daoy Cells, with Condition-Dependent Effect on Steady State Level of MECP21 Transcripts

**Figure 4:**
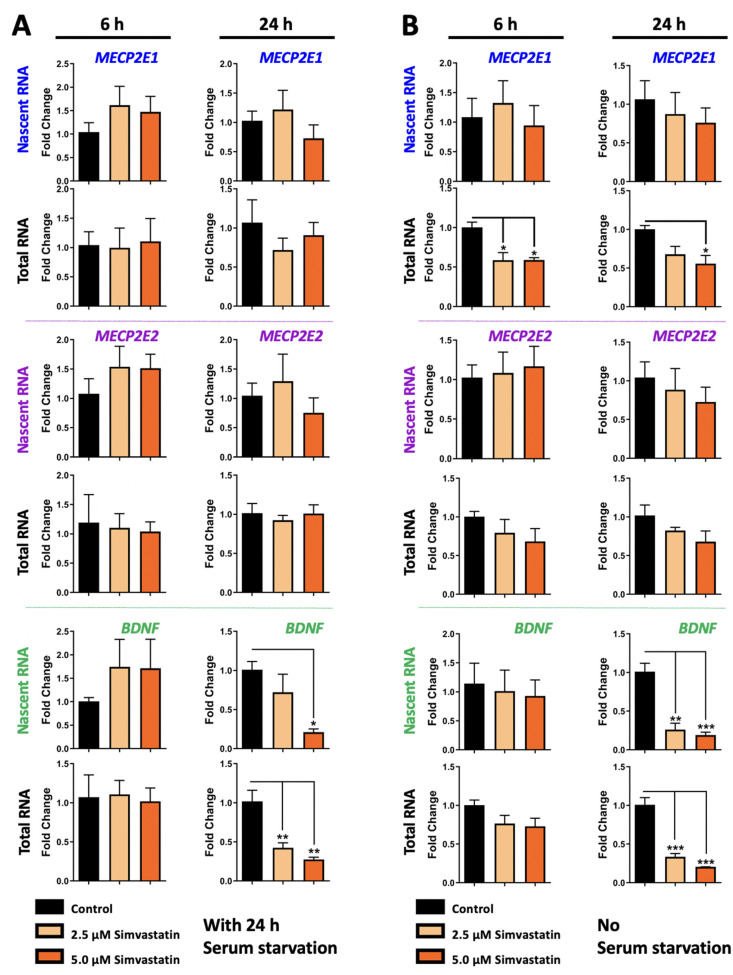
Transcriptional effect of simvastatin on *MECP2E1-E2* and *BDNF* in Daoy cells under the condition with and without serum starvation. (**A**) Daoy cells were serum-starved for 24 h prior to drug treatment with 2.5 and 5.0 μM simvastatin. Samples were collected at 6 and 24 h and gene transcripts were analyzed by RT-PCR for *MECP2E1*, *MECP2E2*, and *BDNF*. C_T_ values were normalized to the housekeeping gene *GAPDH* and fold change was determined relative to the average control. N = 3 ± SEM. Fold change values were analyzed by one-way ANOVA followed by Tukey’s multiple comparisons test, * *p* < 0.05, ** *p* < 0.01, and *** *p* < 0.001. (**B**) Similar to A, but Daoy cells were subjected to the same drug treatment, without prior serum starvation.

To study the role of simvastatin on *MECP2E1/E2-BDNF* transcripts, Daoy cells were treated with two concentrations of simvastatin at 2.5 μM and 5.0 μM. Nascent and steady-state *MECP2E1*, *MECP2E2*, and *BDNF* transcripts are shown in Figure 4A,B. *MECP2E1* and *MECP2E2* transcript levels did not show any statistically significant changes following simvastatin treatments at 6 h or 24 h in condition with serum starvation (Figure 4A, top and middle panels). Nascent and steady-state *BDNF* transcripts were not significantly changed at 6 h (Figure 4A, bottom panels). However, at 24 h, nascent *BDNF* transcripts were significantly decreased in 5.0 μM simvastatin treated cells by approximately 80% (*p* < 0.05). Steady-state *BDNF* transcripts were significantly decreased at 24 h in both 2.5 μM and 5.0 μM simvastatin treated cells by approximately 60% and 70%, respectively (*p* < 0.01) (Figure 4A, bottom panels). Therefore, 5.0 μM simvastatin treatment resulted in reduced *BDNF* transcription after 24 h. Though steady-state *BDNF* was decreased to similar levels by both simvastatin concentrations, nascent *BDNF* only showed statistically significant decreases in the 5.0 μM treatment. Simvastatin at 2.5 μM treatment also appeared to result in a slight decrease in nascent *BDNF*, but this was not statistically significant.

While similar results were obtained for *MECP2E2* in cells without serum starvation (Figure 4B, middle panels), decreased steady state expression of *MECP2E1* was detected at 6 h and 24 h, suggesting a post-transcriptional effect on *MECP2E1*. Similarly, simvastatin reduced *BDNF* transcripts both at the nascent and steady state RNA levels, suggesting transcriptional inhibition of *BDNF* by simvastatin at 24 h. As indicated, nascent *MECP2E1* transcript levels were unchanged in Daoy cells treated with simvastatin in a non-serum starved condition (Figure 4B, top panel), similar to serum starved condition. However, decreases in steady-state *MECP2E1* transcripts were observed in the non-serum starved condition, which had not been observed in serum starved condition. At 6 h, steady state *MECP2E1* was reduced by approximately 40% compared to the controls in Daoy cells treated with either 2.5 μM or 5.0 μM simvastatin (*p* < 0.05). At 24 h, steady-state *MECP2E1* was reduced by approximately 30% compared to controls in Daoy cells treated with 2.5 μM simvastatin, which was not statistically significant, but remained reduced by approximately 40% by 5.0 μM simvastatin (*p* < 0.05). Nascent and steady-state *MECP2E2* transcripts were not significantly altered by simvastatin treatment in the non-serum starved condition (Figure 4B, middle panels). Like the results seen in the serum starved condition, nascent *BDNF* transcripts were significantly reduced at 24 h in cells treated with 2.5 μM and 5.0 μM simvastatin by approximate fold changes of 70–80% for both nascent (*p* < 0.01 and *p* < 0.001) and steady state (*p* < 0.001) transcripts (Figure 4B, bottom panels). This suggests the possibility that simvastatin inhibits *BDNF* expression through transcriptional mechanisms.

### 3.3. Metformin and Simvastatin Do Not Show Additive Transcriptional Effects on MECP2E1/E2-BDNF

**Figure 5:**
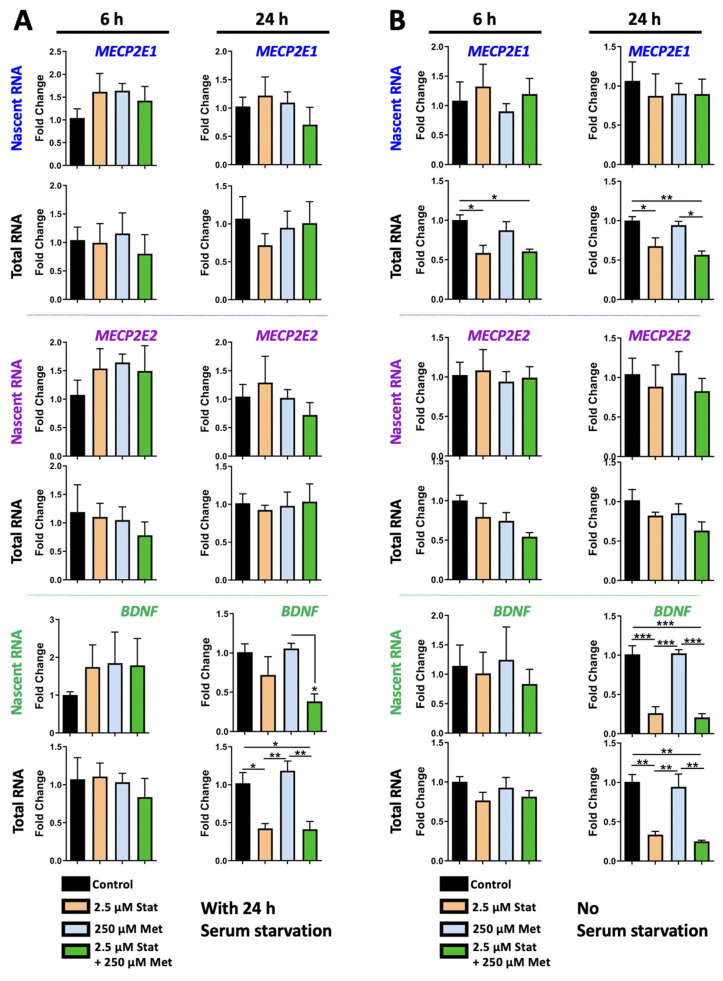
Combined transcriptional effects of simvastatin and metformin on *MECP2E1-E2* and *BDNF* in Daoy cells under the condition with and without serum starvation. (**A**) Daoy cells were serum-starved prior to drug treatment with 2.5 μM simvastatin (Stat), 250 μM metformin (Met), and a combination of 2.5 μM Stat + 250 μM metformin. Samples were collected at 6 and 24 h and gene transcripts (nascent and steady-state) were analyzed by RT-PCR for *MECP2E1*, *MECP2E2*, and *BDNF*. C_T_ values were normalized to the housekeeping gene *GAPDH* and fold change was determined relative to the average control. N = 3 ± SEM. Fold change values were analyzed by one-way ANOVA followed by Tukey’s multiple comparisons test, * *p* < 0.05, ** *p* < 0.01, *** *p* < 0.001. (**B**) Similar to A, but Daoy cells were subjected to the same drug treatment without prior serum starvation.

Combination treatments may be required for MeCP2-related disorders due to the broad impact of *MECP2* mutations. Combining metformin and simvastatin may be of interest in correcting both the glucose and cholesterol metabolism abnormalities seen in RTT patients. The scope of this study was to assess changes in gene expression resulting from treatment with these drugs, and this was also tested for one combination condition. Thus, a combination of 250 μM metformin and 2.5 μM simvastatin was tested for possible synergistic effects of the two drugs under condition with or without serum starvation for 24 h. Daoy cells were treated with a combination of 2.5 μM simvastatin and 250 μM metformin for 6 h and 24 h followed by testing of nascent and steady-state levels of *MECP2E1*, *MECP2E2*, and *BDNF* transcripts by RT-PCR (Figure 5A).

Our results showed that *MECP2E1* and *MECP2E2* transcript levels did not have any statistically significant changes in Daoy cells treated with the combination in serum starved conditions, similar to the individual treatments (Figure 5A, top and middle panels). The effect of the combination treatment on *BDNF* transcripts was similar to the effect of simvastatin treatment alone. Nascent and steady-state *BDNF* transcripts were not significantly changed at 6 h (Figure 5A, bottom panels). Nascent *BDNF* transcripts were reduced at 24 h in cells treated with the combination by approximately 60%, which was statistically significant compared to 250 μM metformin (*p* < 0.05), but not compared to the control. Steady-state *BDNF* transcripts were reduced by about 60% in cells treated with 2.5 μM simvastatin alone as well as the combination, and these decreases were both statistically significant compared to the control (*p* < 0.05) and cells treated with 250 μM metformin (*p* < 0.01) (Figure 5A, bottom panels). The effect of 2.5 μM simvastatin on steady-state *BDNF* transcripts appeared to predominate in the combination treatment.

Analyzing cells under conditions of no serum starvation resulted in the same conclusion for *MECP2E2* (Figure 5B, middle panels), with reduced steady-state transcripts for *MECP2E1* by simvastatin at 6 h and 24 h and inhibition of *BDNF* transcription at 24 h (Figure 5B, top and bottom panels). In the combination treatments of metformin and simvastatin in non-serum starved conditions, *MECP2E1* steady state transcripts were significantly reduced at both 6 h and 24 h in cells treated with 2.5 μM simvastatin and combination of 2.5 μM simvastatin and 250 μM metformin by approximately 50% (*p* < 0.5 and *p* < 0.01) (Figure 5B, top panels). *MECP2E2* transcript levels did not show any statistically significant changes in Daoy cells treated with a combination of the two drugs, similar to individual treatments, although steady state transcripts were reduced approximately 50% at 6- and 24-h, this was not statistically significant (Figure 5B, middle panels). The effect of combination treatment on *BDNF* transcripts was again a significant reduction. Nascent and steady state *BDNF* transcripts were not significantly changed at 6 h (Figure 5B, bottom panels). Nascent *BDNF* transcripts were reduced at 24 h in cells treated with 2.5 μM simvastatin by approximately 30% (*p* < 0.001) and in cells treated with the combination by approximately 20% (*p* < 0.001). These reductions were also statistically significant compared to 250 μM metformin (*p* < 0.001). Steady state *BDNF* transcripts were similarly reduced in cells treated with 2.5 μM simvastatin as well as the combination, and these decreases were both statistically significant compared to the control (*p* < 0.01) and cells treated with 250 μM metformin (*p* < 0.01). Future studies could expand the results to include additional combination treatments of metformin and simvastatin.

### 3.4. Metformin and Simvastatin Treatment Do Not Impact Ribosomal RNA Expression

**Figure 6:**
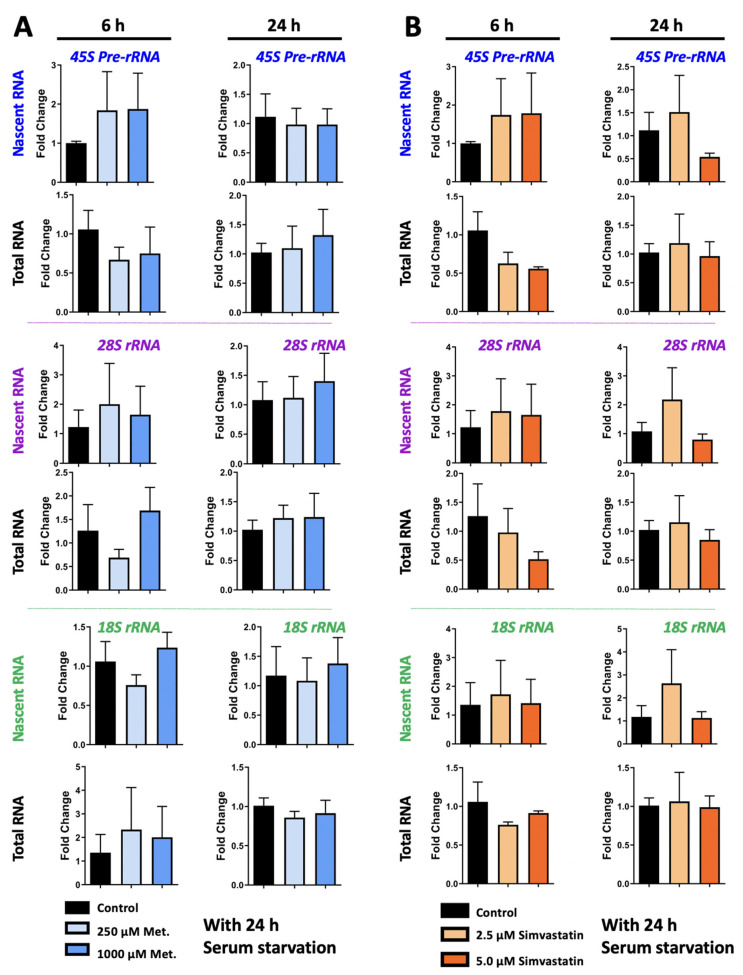
Transcriptional effect of individual metabolic drugs; metformin and simvastatin, on ribosomal *RNA* genes in Daoy cells. Cells were serum-starved for 24 h prior to drug treatment with 250 μM and 1000 μM metformin (**A**), or simvastatin (**B**) at two concentrations of 2.5 and 5.0 μM. Samples were collected at 6 and 24 h and gene expression was analyzed by RT-PCR for *45S pre-rRNA*, *28S rRNA*, and *18S rRNA* transcripts. C_T_ values were normalized to the housekeeping gene *GAPDH* and fold change was determined relative to the average control. N = 3 ± SEM. Fold change values were analyzed by one-way ANOVA followed by Tukey’s multiple comparisons test.

**Figure 7:**
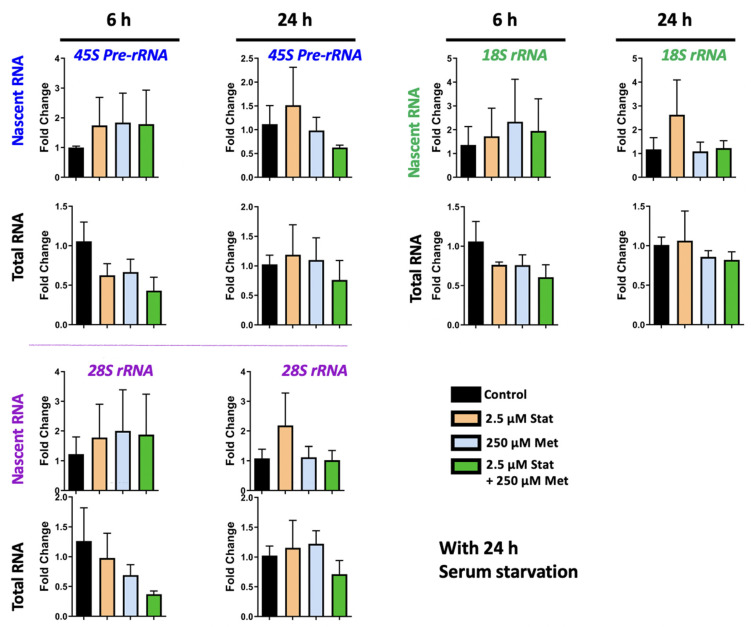
Combined transcriptional effect of simvastatin and metformin on ribosomal *RNA* genes with serum starvation. Daoy cells were serum-starved for 24 h prior to drug treatment with 2.5 μM simvastatin (Stat), 250 μM metformin (Met), and a combination of 2.5 μM Stat + 250 μM metformin. Samples were collected at 6 and 24 h and gene transcripts were analyzed by RT-PCR for *45S pre-rRNA*, *28S rRNA*, and *18S rRNA* transcripts. C_T_ values were normalized to the housekeeping gene *GAPDH* and fold change was determined relative to the average control. N = 3 ± SEM. Fold change values were analyzed by one-way ANOVA followed by Tukey’s multiple comparisons test.

Previous studies from ourselves and others have implicated MeCP2 in regulating *rRNA* transcripts [28,59]. Thus, we evaluated *rRNA* expression in metformin and simvastatin treated Daoy cells. Nascent and steady state *45S pre-rRNA*, *28S rRNA*, and *18S rRNA* transcripts were evaluated in Daoy cells treated with 2.5 μM and 5.0 μM simvastatin, 250 μM and 1000 μM metformin, and the combination treatment of 2.5 μM simvastatin + 250 μM metformin. Two time points of 6 h and 24 h were evaluated. No statistically significant differences in *rRNA* transcripts were observed because of metformin treatment (Figure 6A). There were also no statistically significant differences in *rRNA* transcript levels in Daoy cells treated with simvastatin (Figure 6B) Though a trend of a slight decrease was observed in levels of steady state *rRNA* transcripts at 6 h, these were not statistically significant, and levels appeared similar to the controls by 24 h. No statistically significant differences were observed in Daoy cells treated with a combination of 2.5 μM simvastatin + 250 μM metformin (Figure 7). In evaluating *rRNA* transcripts, high level of variation between biological replicates was observed, and no statistical significance was detected. Overall, these results indicate that simvastatin and metformin treatment at the time points and under these conditions does not impact the *45S rRNA* precursor or processed *28S* and *18S* transcripts.

## 4. Discussion

This study was intended to provide insight into the effects of metformin and simvastatin on the nascent transcription of *MECP2E1*, *MECP2E2*, and *BDNF* in a human brain cell line. The results indicated that simvastatin inhibits *BDNF* transcription, reducing *MECP2E1* steady state transcripts in no serum-starved cells, without any effect on *MECP2E2*. On the other hand, metformin increased transcript levels of *MECP2E1* (in a time-, lot-, and dose-dependent manner) and *BDNF* while decreasing *MECP2E2* transcription. Nascent transcripts of *MECP2E1* and *BDNF* were unchanged by metformin treatment, which indicated that the mechanism by which metformin acts is not directly transcriptional. Future studies could address whether metformin treatment influences the transcript stability of *MECP2E1* and *BDNF*, resulting in the increase in the steady state transcripts. The results of metformin treatment also indicated that an isoform-specific effect is occurring. This highlights the importance of investigating the mechanisms of isoform-specific regulation and determining how they may be relevant to the pathology of Rett Syndrome, *MECP2* Duplication Syndrome, and other MeCP2-associated disorders.

Metformin and simvastatin are both well-established metabolism-modulating drugs, whose effects are suggested to correct the metabolic defects detected in RTT patients. Here, the effects of metformin and simvastatin treatment on *MECP2E1*, *MECP2E2*, *BDNF*, *45S rRNA*, *28S rRNA*, and *18S rRNA* transcription were tested in Daoy cells using nascent RNA analysis. The mechanistic effects of metformin in liver cells are well known, and of simvastatin on cholesterol biosynthesis, but there is limited knowledge of the effects of these drugs in brain cell models.

Metformin and simvastatin cross the blood–brain barrier [35,60]. Metformin has been shown to enhance neurogenesis and spatial memory formation in adult mice through increased phosphorylation of atypical protein kinase C (aPKC) and activation of CREB binding protein (CBP) [61]. Further studies could address whether these signaling molecules are involved in the effects of metformin on *MECP2E1, MECP2E2*, and *BDNF*. Simvastatin promoted neurogenesis in models of traumatic brain injury involving increased BDNF expression, which appears in contradiction to our observation of reduced *BDNF* transcription in Daoy cells treated with simvastatin [37]. This could be a result of the differences between the in vivo and in vitro contexts or differential impact at the transcript and protein levels. A wider range of simvastatin concentrations could also be tested. Further study of the effects of both drugs on DNA methylation, transcription factors regulating the genes of interest, and microRNAs is needed to characterize the role of these drugs in brain cell models. In humans, the *BDNF* gene has eleven exons and nine functional promoters with the last exon containing the coding region [62]. Regulation is complex with many transcription factors at play [63], which could potentially be influenced by drug treatment.

In our studies, metformin induced *BDNF*, while simvastatin inhibited it. These effects were detectable after 24 h. Whether or not either drug acts through mediatory mechanisms that result in indirect regulation of *BDNF* transcription or transcript stability is unclear. BDNF is known to be influenced by inflammation-related pathways [64], thus, we cannot exclude the possibility that metformin induction of *BDNF* might be indirect. Similarly, inhibition of *BDNF* by simvastatin could either be direct or indirect through different regulatory mechanisms that warrant further investigations.

Daoy medulloblastoma cells are commonly used for studies on MeCP2 regulation and function [65,66]. Our results were in experimental conditions in cells either with or without serum starvation. Serum deprivation is a common method to synchronize cells prior to drug treatment and is commonly used to bring the cell populations to a similar metabolic state in the same stage of the cell cycle [67]. This is expected to result in consistent effect among the cells. In our study, the transcript levels detected in cells that did not undergo serum starvation prior to drug treatment showed some differences with those that were serum starved.

The selected doses for the drugs in in vitro studies may commonly differ from what is detectable in patients. Regarding metformin, type 2 diabetes patients receiving an 850 mg metformin dose have a plasma metformin concentration of approximately 8–24 μM 3 h after the dose. Caution is required with increasing metformin dosages since patients with plasma metformin concentrations of 150–820 μM may develop lactic acidosis due to increased plasma lactate levels. Plasma concentrations of approximately 2.5 mg/L, or 20 μM are generally considered to be safe [68]. In our study, Daoy cells were treated with 250, 1000, and 2000 μM metformin, which are higher compared to what is recommended for patient plasma levels. However, the purpose of this study was to evaluate the transcriptional effects of metformin on gene transcripts in vitro over a short time. In agreement with our approach, concentrations of metformin in this range have previously been used in vitro to analyze the impact of metformin on cell signaling molecules [61,69]. Future studies could investigate the effects of prolonged metformin treatment at lower concentrations.

In addition to neurodevelopmental disorders such as Fragile X syndrome, clinical studies have shown that metformin treatment also lowers the probability of Parkinson’s disease in type 2 diabetes patients [70]. The neuroprotective effects of metformin were also seen in mouse models of Parkinson’s disease with increased BDNF protein levels observed in metformin-treated mice [71,72]. Metformin was also found to enhance neurogenesis and spatial memory formation in mice [71].

For simvastatin treatment in Daoy cells, we selected doses based on a recent study from our own lab [73]. Simvastatin concentrations of 2.5 and 5.0 μM were selected here for a 24 h treatment. This is a greater concentration than what is typically found in the plasma of patients taking statins, which tends to peak at a wide nanomolar range of 6 nM to 80 nM. In vitro studies have commonly employed statins in the micromolar range [74,75]. Our results showed that 2.5 and 5.0 μM simvastatin treatment significantly inhibited *BDNF* transcription in Daoy cells, but did not significantly impact *MECP2E2* transcripts. The effects of simvastatin on *MECP2E1* showed some variation between experimental conditions of the presence or absence of serum starvation prior to drug treatment. Cells that were not serum starved prior to drug treatment showed some decrease in *MECP2E1* and *MECP2E2* transcripts, indicating the metabolic state of cells may impact the effect of simvastatin on transcript levels. Simvastatin treatment of Daoy cells has also been tested in applications as a cancer therapeutic. As cancer cells, Daoy cells may respond differently to cholesterol inhibition than perhaps a neuronal in vitro model or an in vivo model would show. Toxic effects of simvastatin have not been observed previously in other mouse models of neurological disorders, and it may be most ideal to test this drug in the in vivo model of RTT.

Our approach was a side-by-side comparison of the transcriptional effect of these drugs on nascent RNA versus steady state total RNA transcripts. In vitro studies that used this technique include imaging of the nascent RNA production in cultured NIH-3T3 cells [51] as well as in dendrites of cultured hippocampal neurons [76]. Targeted gene transcript analysis has also been reported in vitro for *NEAT1* transcripts in HeLa cells [77] and to study *MECP2* post-transcriptional regulation in a human embryonic stem cell (hESC) differentiation model of neurodevelopment [78]. In vivo studies have been performed by injecting EU into mice and zebrafish [51,76].

Taken together, this current study would contribute to our knowledge about the potential application of metformin for transcriptional regulation of *MECP2E1/E2-BDNF* homeostasis and provides important insight toward future therapeutic studies for RTT.

Since treatment of RTT patients with exogenous BDNF is not feasible due to its low blood–brain barrier permeability, small molecule drug treatments that do pass the BBB and stimulate BDNF expression are of interest. Fingolimod has been used to indirectly stimulate BDNF metabolism, improving locomotion in *Mecp2*-null mice and increasing the volume of the striatum, an important region of BDNF transport and function [79]. Ampakine treatment increased BDNF levels through stimulating neuronal activation and improved breathing patterns in *Mecp2-*null mice [80]. Agonists of the TrkB receptor, the receptor for BDNF, also improve breathing patterns [81,82]. In these studies, treatment had to be initiated before the appearance of first symptoms in order to be efficacious, but usually RTT is diagnosed long after the symptoms have begun. Treatment with FK506, a calcineurin inhibitor, can be initiated after the symptoms appear and improves the transportation of BDNF between brain regions, improves lifespan, motor strength and coordination, and exploratory behavior, and reduced the frequency of apneas in *Mecp2-*null mice [83]. RTT patients may benefit from a combination treatment with a drug that induces MeCP2 and/or BDNF expression along with a drug that corrects BDNF transport.

To our knowledge, this study is the first to show that metformin induces *MECP2E1* and *BDNF* transcripts while transcriptionally inhibits *MECP2E2* transcripts. Perhaps this is part of the mechanism by which metformin enhances neurogenesis in mice [71]. The effects of metformin treatment on transcript levels were time- and concentration-dependent. In Daoy cells, changes in *MECP2E1*, *MECP2E2*, and *BDNF* transcripts were all seen at 48 h in cells treated with 2000 μM metformin. There was a slight increase in steady-state *MECP2E1* transcripts and approximately 2-fold increase in *BDNF* transcripts, but these were not changed at the nascent RNA level. *MECP2E2* transcripts were decreased by metformin treatment at the nascent and steady-state levels. Further studies are required to determine how metformin affects transcripts at different levels of regulation, and how it can produce MeCP2 isoform-specific regulation mechanisms. It is still unknown whether these transcript changes are also translated to the level of protein.

Further in vivo studies of metformin in RTT models should evaluate the effects of metformin on glucose metabolism and whether increases in *MECP2E1* and *BDNF* are observed in vivo. Decreases in *MECP2E2*, which were observed in this study, may have potential application where its overexpression is detected in disease states. For example, in the case of *MECP2* Duplication Syndrome, metformin may be of interest if it is capable of somewhat normalizing MeCP2 levels. In addition, the combination of metformin and simvastatin in vivo can be tested to target both glucose and cholesterol metabolism abnormalities.

## 5. Limitations of the Study

One limitation of this study would be the effects of simvastatin and metformin treatments on MeCP2 and BDNF protein levels at the nascent protein and steady-state levels.

## 6. Conclusions

In conclusion, *BDNF* transcript levels were shown to be targeted by both metformin and simvastatin in a Daoy cell model, although in opposite directions. While simvastatin did not significantly alter the *MECP2E2* isoform, metformin showed an isoform-specific inhibition of *MECP2E2*. Metformin was also capable of inducing *MECP2E1*, although this effect in our hands was dependent on the lot of the drug. The true efficacy of these drugs could further be evaluated by in vivo studies using Rett Syndrome or MDS transgenic mice. Metformin treatment resulted in significantly increased *BDNF* transcripts in Daoy cells, which along with its previously demonstrated function of enhancing neurogenesis indicate it to be considered as a promising therapeutic for RTT Syndrome.

## Figures and Tables

**Table 1:** Primer sequences used for qRT-PCR in this study.

Primer Name		Sequence	References
*GAPDH*	Forward	5′-CCACTCCTCCACCTTTGAC-3′	[26,53]
	Reverse	5′-ACCCTGTTGCTGTAGCCA-3′	
*MECP2E1*	Forward	5′-AGGAGAGACTGGAAGAAAAGTC-3′	[26,54]
	Reverse	5′-CTTGAGGGGTTTGTCCTTGA-3′	
*MECP2E2*	Forward	5′-CTCACCAGTTCCTGCTTTGATGT-3′	[26,54]
	Reverse	5′-CTTGAGGGGTTTGTCCTTGA-3′	
*BDNF*	Forward	5′-TAACGGCGGCAGACAAAAAGA-3′	[26,55]
	Reverse	5′-GAAGTATTGCTTCAGTTGGCCT-3′	
*45S rRNA*	Forward	5′-CTCCGTTATGGTAGCGCTGC-3′	[28,56]
	Reverse	5′-GCGGAACCCTCGCTTCTC-3′	
*28S rRNA*	Forward	5′-AGAGGTAAACGGGTGGGGTC-3′	[28,57]
	Reverse	5′-GGGGTCGGGAGGAACGG-3′	
*18S rRNA*	Forward	5′-GATGGTAGTCGCCGTGCC-3′	[28,57]
	Reverse	5′-GCCTGCTGCCTTCCTTGG-3′	

## Data Availability

All the relevant data for this study are presented in the article.

## References

[1] Amir R.E., Van den Veyver I.B., Wan M., Tran C.Q., Francke U., Zoghbi H.Y. (1999). Rett syndrome is caused by mutations in X-linked *MECP2*, encoding methyl-CpG-binding protein 2. Nat. Genet..

[2] Liyanage V.R., Jarmasz J.S., Murugeshan N., Del Bigio M.R., Rastegar M., Davie J.R. (2014). DNA modifications: Function and applications in normal and disease States. Biology.

[3] Delcuve G.P., Rastegar M., Davie J.R. (2009). Epigenetic control. J. Cell Physiol..

[4] Jarmasz J.S., Stirton H., Davie J.R., Del Bigio M.R. (2019). DNA methylation and histone post-translational modification stability in post-mortem brain tissue. Clin. Epigenet..

[5] Liyanage V.R., Rastegar M. (2014). Rett syndrome and MeCP2. Neuromol. Med..

[6] Rastegar M. (2017). Epigenetics and Cerebellar Neurodevelopmental Disorders. Development of the Cerebellum from Molecular Aspects to Diseases.

[7] Zachariah R.M., Rastegar M. (2012). Linking epigenetics to human disease and Rett syndrome: The emerging novel and challenging concepts in MeCP2 research. Neural Plast..

[8] Moore L.D., Le T. (2013). Fan GDNA methylation its basic function. Neuropsychopharmacology.

[9] Chen L., Chen K., Lavery L.A., Baker S.A., Shaw C.A., Li W., Zoghbi H.Y. (2015). MeCP2 binds to non-CG methylated DNA as neurons mature, influencing transcription and the timing of onset for Rett syndrome. Proc. Natl. Acad. Sci. USA.

[10] Rodriguez-Casariego J.A., Ladd M.C., Shantz A.A., Lopes C., Cheema M.S., Kim B., Roberts S.B., Fourqurean J.W., Ausio J., Burkepile D.E. (2018). Coral epigenetic responses to nutrient stress: Histone H2A. X phosphorylation dynamics and DNA methylation in the staghorn coral Acropora cervicornis. Ecol. Evol..

[11] Olson C.O., Zachariah R.M., Ezeonwuka C.D., Liyanage V.R., Rastegar M. (2014). Brain region-specific expression of MeCP2 isoforms correlates with DNA methylation within *Mecp2* regulatory elements. PLoS ONE.

[12] Liyanage V.R.B., Olson C.O., Zachariah R.M., Davie J.R., Rastegar M. (2019). DNA Methylation Contributes to the Differential Expression Levels of *Mecp2* in Male Mice Neurons and Astrocytes. Int. J. Mol. Sci..

[13] Yasui D.H., Gonzales M.L., Aflatooni J.O., Crary F.K., Hu D.J., Gavino B.J., Golub M.S., Vincent J.B., Carolyn Schanen N., Olson C.O. (2014). Mice with an isoform-ablating *Mecp2* exon 1 mutation recapitulate the neurologic deficits of Rett syndrome. Hum. Mol. Genet..

[14] Martinez de Paz A., Khajavi L., Martin H., Claveria-Gimeno R., Tom Dieck S., Cheema M.S., Sanchez-Mut J.V., Moksa M.M., Carles A., Brodie N.I. (2019). MeCP2-E1 isoform is a dynamically expressed, weakly DNA-bound protein with different protein and DNA interactions compared to MeCP2-E2. Epigenet. Chromatin..

[15] Ezeonwuka C.D., Rastegar M. (2014). MeCP2-Related Diseases and Animal Models. Diseases.

[16] Mnatzakanian G.N., Lohi H., Munteanu I., Alfred S.E., Yamada T., MacLeod P.J., Jones J.R., Scherer S.W., Schanen N.C., Friez M.J. (2004). A previously unidentified *MECP2* open reading frame defines a new protein isoform relevant to Rett syndrome. Nat. Genet..

[17] Rastegar M., Hotta A., Pasceri P., Makarem M., Cheung A.Y., Elliott S., Park K.J., Adachi M., Jones F.S., Clarke I.D. (2009). *MECP2* isoform-specific vectors with regulated expression for Rett syndrome gene therapy. PLoS ONE.

[18] Zachariah R.M., Olson C.O., Ezeonwuka C., Rastegar M. (2012). Novel MeCP2 isoform-specific antibody reveals the endogenous MeCP2E1 expression in murine brain, primary neurons and astrocytes. PLoS ONE.

[19] Pejhan S., Rastegar M. (2021). Role of DNA Methyl-CpG-Binding Protein MeCP2 in Rett Syndrome Pathobiology and Mechanism of Disease. Biomolecules.

[20] Djuric U., Cheung A.Y., Zhang W., Mok R.S., Lai W., Piekna A., Hendry J.A., Ross P.J., Pasceri P., Kim D.S. (2015). MECP2e1 isoform mutation affects the form and function of neurons derived from Rett syndrome patient iPS cells. Neurobiol. Dis..

[21] Liyanage V.R., Zachariah R.M., Davie J.R., Rastegar M. (2015). Ethanol deregulates *Mecp2*/MeCP2 in differentiating neural stem cells via interplay between 5-methylcytosine and 5-hydroxymethylcytosine at the Mecp2 regulatory elements. Exp. Neurol..

[22] Liyanage V.R., Zachariah R.M., Rastegar M. (2013). Decitabine alters the expression of *Mecp2* isoforms via dynamic DNA methylation at the *Mecp2* regulatory elements in neural stem cells. Mol. Autism..

[23] Xu W., Liyanage V.R.B., MacAulay A., Levy R.D., Curtis K., Olson C.O., Zachariah R.M., Amiri S., Buist M., Hicks G.G. (2019). Genome-Wide Transcriptome Landscape of Embryonic Brain-Derived Neural Stem Cells Exposed to Alcohol with Strain-Specific Cross-Examination in BL6 and CD1 Mice. Sci. Rep..

[24] Miranda-Lourenco C., Duarte S.T., Palminha C., Gaspar C., Rodrigues T.M., Magalhaes-Cardoso T., Rei N., Colino-Oliveira M., Gomes R., Ferreira S. (2020). Impairment of adenosinergic system in Rett syndrome: Novel therapeutic target to boost BDNF signalling. Neurobiol. Dis..

[25] Dechant G., Barde Y.A. (2002). The neurotrophin receptor p75(NTR): Novel functions and implications for diseases of the nervous system. Nat. Neurosci..

[26] Pejhan S., Del Bigio M.R., Rastegar M. (2020). The MeCP2E1/E2-BDNF- miR132 Homeostasis Regulatory Network Is Region-Dependent in the Human Brain and Is Impaired in Rett Syndrome Patients. Front. Cell Dev. Biol..

[27] Pejhan S., Siu V.M., Ang L.C., Del Bigio M.R., Rastegar M. (2020). Differential brain region-specific expression of MeCP2 and BDNF in Rett Syndrome patients: A distinct grey-white matter variation. Neuropathol. Appl. Neurobiol..

[28] Olson C.O., Pejhan S., Kroft D., Sheikholeslami K., Fuss D., Buist M., Ali Sher A., Del Bigio M.R., Sztainberg Y., Siu V.M. (2018). *MECP2* Mutation Interrupts Nucleolin-mTOR-P70S6K Signaling in Rett Syndrome Patients. Front. Genet..

[29] Good K.V., Vincent J.B., Ausio J. (2021). MeCP2: The Genetic Driver of Rett Syndrome Epigenetics. Front. Genet..

[30] Sharifi O., Yasui D.H. (2021). The Molecular Functions of MeCP2 in Rett Syndrome Pathology. Front. Genet..

[31] Kyle S.M., Vashi N., Justice M.J. (2018). Rett syndrome: A neurological disorder with metabolic components. Open Biol..

[32] Buchovecky C.M., Turley S.D., Brown H.M., Kyle S.M., McDonald J.G., Liu B., Pieper A.A., Huang W., Katz D.M., Russell D.W. (2013). A suppressor screen in *Mecp2* mutant mice implicates cholesterol metabolism in Rett syndrome. Nat. Genet..

[33] Justice M.J., Buchovecky C.M., Kyle S.M., Djukic A. (2013). A role for metabolism in Rett syndrome pathogenesis: New clinical findings and potential treatment targets. Rare Dis..

[34] Segatto M., Trapani L., Di Tunno I., Sticozzi C., Valacchi G., Hayek J., Pallottini V. (2014). Cholesterol metabolism is altered in Rett syndrome: A study on plasma and primary cultured fibroblasts derived from patients. PLoS ONE.

[35] McFarland A.J., Anoopkumar-Dukie S., Arora D.S., Grant G.D., McDermott C.M., Perkins A.V., Davey A.K. (2014). Molecular mechanisms underlying the effects of statins in the central nervous system. Int. J. Mol. Sci..

[36] Orth M., Bellosta S. (2012). Cholesterol: Its regulation and role in central nervous system disorders. Cholesterol.

[37] Wu H., Lu D., Jiang H., Xiong Y., Qu C., Li B., Mahmood A., Zhou D., Chopp M. (2008). Simvastatin-mediated upregulation of VEGF and BDNF, activation of the PI3K/Akt pathway, and increase of neurogenesis are associated with therapeutic improvement after traumatic brain injury. J. Neurotrauma.

[38] Howell J.J., Hellberg K., Turner M., Talbott G., Kolar M.J., Ross D.S., Hoxhaj G., Saghatelian A., Shaw R.J., Manning B.D. (2017). Metformin Inhibits Hepatic mTORC1 Signaling via Dose-Dependent Mechanisms Involving AMPK and the TSC Complex. Cell Metab..

[39] Rena G., Hardie D.G., Pearson E.R. (2017). The mechanisms of action of metformin. Diabetologia.

[40] Gantois I., Khoutorsky A., Popic J., Aguilar-Valles A., Freemantle E., Cao R., Sharma V., Pooters T., Nagpal A., Skalecka A. (2017). Metformin ameliorates core deficits in a mouse model of fragile X syndrome. Nat. Med..

[41] Gantois I., Popic J., Khoutorsky A., Sonenberg N. (2019). Metformin for Treatment of Fragile X Syndrome and Other Neurological Disorders. Annu. Rev. Med..

[42] Garfunkel D., Anagnostou E.A., Aman M.G., Handen B.L., Sanders K.B., Macklin E.A., Chan J., Veenstra-VanderWeele J. (2019). Pharmacogenetics of Metformin for Medication-Induced Weight Gain in Autism Spectrum Disorder. J. Child Adolesc. Psychopharmacol..

[43] Calkin C., McClelland C., Cairns K., Kamintsky L., Friedman A. (2021). Insulin Resistance and Blood-Brain Barrier Dysfunction Underlie Neuroprogression in Bipolar Disorder. Front. Psychiatry.

[44] Sommer I.E., Gangadin S.S., de Witte L.D., Koops S., van Baal C., Bahn S., Drexhage H., van Haren N.E.M., Veling W., Bruggeman R. (2021). Simvastatin Augmentation for Patients with Early-Phase Schizophrenia-Spectrum Disorders: A Double-Blind, Randomized Placebo-Controlled Trial. Schizophr. Bull..

[45] Protic D., Aydin E.Y., Tassone F., Tan M.M., Hagerman R.J., Schneider A. (2019). Cognitive and behavioral improvement in adults with fragile X syndrome treated with metformin-two cases. Mol. Genet. Genomic. Med..

[46] Watanabe K., Asano D., Ushikubo H., Morita A., Mori A., Sakamoto K., Ishii K., Nakahara T. (2021). Metformin Protects against NMDA-Induced Retinal Injury through the MEK/ERK Signaling Pathway in Rats. Int. J. Mol. Sci..

[47] Du R.W., Bu W.G. (2021). Simvastatin Prevents Neurodegeneration in the MPTP Mouse Model of Parkinson’s Disease via Inhibition of A1 Reactive Astrocytes. Neuroimmunomodulation.

[48] Lamonica J.M., Kwon D.Y., Goffin D., Fenik P., Johnson B.S., Cui Y., Guo H., Veasey S., Zhou Z. (2017). Elevating expression of MeCP2 T158M rescues DNA binding and Rett syndrome-like phenotypes. J. Clin. Investig..

[49] Barber B.A., Liyanage V.R., Zachariah R.M., Olson C.O., Bailey M.A., Rastegar M. (2013). Dynamic expression of MEIS1 homeoprotein in E14.5 forebrain and differentiated forebrain-derived neural stem cells. Ann. Anat..

[50] Russo J., Heck A.M., Wilusz J., Wilusz C.J. (2017). Metabolic labeling and recovery of nascent RNA to accurately quantify mRNA stability. Methods.

[51] Jao C.Y., Salic A. (2008). Exploring RNA transcription and turnover in vivo by using click chemistry. Proc. Natl. Acad. Sci. USA.

[52] Amiri S., Davie J.R., Rastegar M. (2020). Chronic Ethanol Exposure Alters DNA Methylation in Neural Stem Cells: Role of Mouse Strain and Sex. Mol. Neurobiol..

[53] Zheng S.Y., Hou J.Y., Zhao J., Jiang D., Ge J.F., Chen S. (2012). Clinical outcomes of downregulation of E-cadherin gene expression in non-small cell lung cancer. Asian Pac. J. Cancer Prev..

[54] Sztainberg Y., Chen H.M., Swann J.W., Hao S., Tang B., Wu Z., Tang J., Wan Y.W., Liu Z., Rigo F. (2015). Reversal of phenotypes in *MECP2* duplication mice using genetic rescue or antisense oligonucleotides. Nature.

[55] Zuccato C., Marullo M., Vitali B., Tarditi A., Mariotti C., Valenza M., Lahiri N., Wild E.J., Sassone J., Ciammola A. (2011). Brain-derived neurotrophic factor in patients with Huntington’s disease. PLoS ONE.

[56] Stimpson K.M., Sullivan L.L., Kuo M.E., Sullivan B.A. (2014). Nucleolar organization, ribosomal DNA array stability, and acrocentric chromosome integrity are linked to telomere function. PLoS ONE.

[57] Uemura M., Zheng Q., Koh C.M., Nelson W.G., Yegnasubramanian S., De Marzo A.M. (2012). Overexpression of ribosomal RNA in prostate cancer is common but not linked to rDNA promoter hypomethylation. Oncogene.

[58] Vippagunta R.R., LoBrutto R., Pan C., Lakshman J.P. (2010). Investigation of Metformin HCl lot-to-lot variation on flowability differences exhibited during drug product processing. J. Pharm. Sci..

[59] Ghoshal K., Majumder S., Datta J., Motiwala T., Bai S., Sharma S.M., Frankel W., Jacob S.T. (2004). Role of human ribosomal RNA (rRNA) promoter methylation and of methyl-CpG-binding protein MBD2 in the suppression of rRNA gene expression. J. Biol. Chem..

[60] Labuzek K., Suchy D., Gabryel B., Bielecka A., Liber S., Okopien B. (2010). Quantification of metformin by the HPLC method in brain regions, cerebrospinal fluid and plasma of rats treated with lipopolysaccharide. Pharmacol. Rep..

[61] Wang J., Gallagher D., DeVito L.M., Cancino G.I., Tsui D., He L., Keller G.M., Frankland P.W., Kaplan D.R., Miller F.D. (2012). Metformin activates an atypical PKC-CBP pathway to promote neurogenesis and enhance spatial memory formation. Cell Stem Cell.

[62] Pruunsild P., Kazantseva A., Aid T., Palm K., Timmusk T. (2007). Dissecting the human BDNF locus: Bidirectional transcription, complex splicing, and multiple promoters. Genomics.

[63] West A.E., Pruunsild P., Timmusk T. (2014). Neurotrophins: Transcription and translation. Handb. Exp. Pharmacol..

[64] Zhang J.C., Yao W., Hashimoto K. (2016). Brain-derived Neurotrophic Factor (BDNF)-TrkB Signaling in Inflammation-related Depression and Potential Therapeutic Targets. Curr. Neuropharmacol..

[65] Han K., Gennarino V.A., Lee Y., Pang K., Hashimoto-Torii K., Choufani S., Raju C.S., Oldham M.C., Weksberg R., Rakic P. (2013). Human-specific regulation of MeCP2 levels in fetal brains by microRNA miR-483–5p. Genes. Dev..

[66] Lombardi L.M., Zaghlula M., Sztainberg Y., Baker S.A., Klisch T.J., Tang A.A., Huang E.J., Zoghbi H.Y. (2017). An RNA interference screen identifies druggable regulators of MeCP2 stability. Sci. Transl. Med..

[67] Cooper S. (2003). Reappraisal of serum starvation, the restriction point, G0, and G1 phase arrest points. FASEB J..

[68] Graham G.G., Punt J., Arora M., Day R.O., Doogue M.P., Duong J.K., Furlong T.J., Greenfield J.R., Greenup L.C., Kirkpatrick C.M. (2011). Clinical pharmacokinetics of metformin. Clin. Pharmacokinet..

[69] Blandino G., Valerio M., Cioce M., Mori F., Casadei L., Pulito C., Sacconi A., Biagioni F., Cortese G., Galanti S. (2012). Metformin elicits anticancer effects through the sequential modulation of DICER and c-MYC. Nat. Commun..

[70] Wahlqvist M.L., Lee M.S., Hsu C.C., Chuang S.Y., Lee J.T., Tsai H.N. (2012). Metformin-inclusive sulfonylurea therapy reduces the risk of Parkinson’s disease occurring with Type 2 diabetes in a Taiwanese population cohort. Parkinsonism Relat. Disord..

[71] Patil S.P., Jain P.D., Ghumatkar P.J., Tambe R., Sathaye S. (2014). Neuroprotective effect of metformin in MPTP-induced Parkinson’s disease in mice. Neuroscience.

[72] Katila N., Bhurtel S., Shadfar S., Srivastav S., Neupane S., Ojha U., Jeong G.S., Choi D.Y. (2017). Metformin lowers alpha-synuclein phosphorylation and upregulates neurotrophic factor in the MPTP mouse model of Parkinson’s disease. Neuropharmacology.

[73] Sheikholeslami K., Ali Sher A., Lockman S., Kroft D., Ganjibakhsh M., Nejati-Koshki K., Shojaei S., Ghavami S., Rastegar M. (2019). Simvastatin Induces Apoptosis in Medulloblastoma Brain Tumor Cells via Mevalonate Cascade Prenylation Substrates. Cancers.

[74] DeGorter M.K., Tirona R.G., Schwarz U.I., Choi Y.H., Dresser G.K., Suskin N., Myers K., Zou G., Iwuchukwu O., Wei W.Q. (2013). Clinical and pharmacogenetic predictors of circulating atorvastatin and rosuvastatin concentrations in routine clinical care. Circ. Cardiovasc. Genet..

[75] Bjorkhem-Bergman L., Lindh J.D., Bergman P. (2011). What is a relevant statin concentration in cell experiments claiming pleiotropic effects?. Br. J. Clin. Pharmacol..

[76] Akbalik G., Langebeck-Jensen K., Tushev G., Sambandan S., Rinne J., Epstein I., Cajigas I., Vlatkovic I., Schuman E.M. (2017). Visualization of newly synthesized neuronal RNA in vitro and in vivo using click-chemistry. RNA Biol..

[77] Hirose T., Virnicchi G., Tanigawa A., Naganuma T., Li R., Kimura H., Yokoi T., Nakagawa S., Benard M., Fox A.H. (2014). NEAT1 long noncoding RNA regulates transcription via protein sequestration within subnuclear bodies. Mol. Biol. Cell.

[78] Rodrigues D.C., Kim D.S., Yang G., Zaslavsky K., Ha K.C., Mok R.S., Ross P.J., Zhao M., Piekna A., Wei W. (2016). *MECP2* Is Post-transcriptionally Regulated during Human Neurodevelopment by Combinatorial Action of RNA-Binding Proteins and miRNAs. Cell Rep..

[79] Deogracias R., Yazdani M., Dekkers M.P., Guy J., Ionescu M.C., Vogt K.E., Barde Y.A. (2012). Fingolimod, a sphingosine-1 phosphate receptor modulator, increases BDNF levels and improves symptoms of a mouse model of Rett syndrome. Proc. Natl. Acad. Sci. USA.

[80] Ogier M., Wang H., Hong E., Wang Q., Greenberg M.E., Katz D.M. (2007). Brain-derived neurotrophic factor expression and respiratory function improve after ampakine treatment in a mouse model of Rett syndrome. J. Neurosci..

[81] Johnson R.A., Lam M., Punzo A.M., Li H., Lin B.R., Ye K., Mitchell G.S., Chang Q. (2012). 7,8-dihydroxyflavone exhibits therapeutic efficacy in a mouse model of Rett syndrome. J. Appl. Physiol. (1985).

[82] Schmid D.A., Yang T., Ogier M., Adams I., Mirakhur Y., Wang Q., Massa S.M., Longo F.M., Katz D.M. (2012). A TrkB small molecule partial agonist rescues TrkB phosphorylation deficits and improves respiratory function in a mouse model of Rett syndrome. J. Neurosci..

[83] Ehinger Y., Bruyere J., Panayotis N., Abada Y.S., Borloz E., Matagne V., Scaramuzzino C., Vitet H., Delatour B., Saidi L. (2020). Huntingtin phosphorylation governs BDNF homeostasis and improves the phenotype of *Mecp2* knockout mice. EMBO Mol. Med..

